# Thermal, mechanical investigation and neutron shielding analysis for Gd-MOF/polyimide materials†

**DOI:** 10.1039/d1ra07500d

**Published:** 2021-12-22

**Authors:** Chen Hu, Qunying Huang, Yutao Zhai

**Affiliations:** Institute of Nuclear Energy Safety Technology, Hefei Institutes of Physical Science, Chinese Academy of Sciences Hefei Anhui 230031 China; University of Science and Technology of China Hefei Anhui 230027 China hu2017@mail.ustc.edu.cn

## Abstract

None of the currently commercialized shielding materials in Generation IV nuclear energy systems are satisfactory in their performance. Developing a candidate neutron shielding material with good heat resistance and high strength is a challenging task. In this work, various gadolinium metal–organic frameworks (Gd-MOFs) with obvious advantages, such as porous structures, organic surfaces and strong neutron-absorbing nuclei, were synthesized to constrain polyimide (PI) chains. A series of Gd-MOF/PI conjugates were subsequently assessed for their thermal stability, mechanical properties and neutron shielding performance. The increase of the Gd-MOF content improved the thermal neutron shielding ability but slightly reduced the fast neutron shielding ability. Compared with those of pure PI, the Gd-MOF/PI films demonstrate a higher glass transition temperature (*T*_g_), which is considered the gold standard of engineering plastics. It was also observed that the tensile strength directly correlates with the Gd-MOF content, which continuously increases until a maximum is reached, and then subsequently decreases. Furthermore, the high-temperature tensile test showed that these tunable Gd-MOF/PI films are intact and robust, indicating their potential application for neutron shielding materials in Generation IV nuclear energy systems.

## Abstract 2

None of the currently commercialized shielding materials in Generation IV nuclear energy systems are satisfactory in their performance.

## Graphical abstract

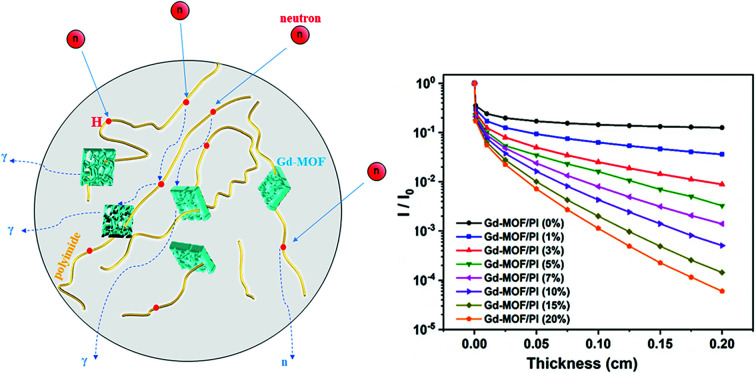

## 1. Introduction

Nuclear technology can revolutionize the energy supply and serve in nuclear power plants and nuclear medicine therapy to benefit manufacturing and surgical procedures. Unwanted exposure to nuclear radiation in nuclear plants or health care facilities has fatally negative effects on the service life of electronic components and the health of the public.^1^ Uncharged neutrons can easily pass through tissues and cause ionization, which is hazardous to health and remains a major challenge in the field of radiation shielding. Neutron shielding is achieved primarily by neutron scattering and adsorption.^2,3^ Due to the high density of electrons in hydrogen-rich polyethylene (PE),^4^ PE has large scattering power and can be utilized as an effective neutron moderator.^5^ Previous investigations have also revealed that some chemical elements, including boron (B) and gadolinium (Gd), are good thermal neutron absorbers that have relatively high thermal neutron absorption cross-sections of 3835 barns for ^10^B, 62 540 barns for ^155^Gd and 255 000 barns for ^157^Gd.^6^ Boron compounds, including boron carbide (B_4_C) and boron nitride (BN),^7^ have widely been used as neutron shielding materials. Moreover, as shown in Fig. S1,† Gd has a much higher neutron absorption cross-section than B.

Many studies have been performed on several neutron shielding materials, and the properties of these materials have been greatly improved in the past decade. Neutron shielding materials can be broadly classified by their components, traditional metals (or ceramics) and lightweight, low-cost polymer composites. The most commonly used traditional materials are boron-containing stainless steel^8^ and boron carbide-aluminum composites,^9^ though their heavy weight, high cost and difficult processing limit further engineering applications in wearable protective devices and miniaturized nuclear plants. PE, epoxy (EP),^10^ polyurethane (PU),^11^ ethylene propylene diene monomer (EPDM)^12^ and boron-containing polyimide (PI)^13^ are widely used in miniaturized and mobile nuclear energy systems. Despite their nontoxicity, good radiation resistance, and better performance than metals (or ceramics) in terms of their elastic nature, low cost and good flexibility,^14,15^ the addition of second phase fillers may destroy the interface compatibility of the polymer composites and surface functionalization of the fillers, leading to high production costs.

MOFs^16^ are framework compounds comprising metallic ion centers connected by organic linkers to create one-, two- and three-dimensional porous frameworks with plentiful physicochemical properties, low densities and adjustable pore volumes and surface areas.^17^ Because they possess such abundant functions, MOFs are highly attractive for application in various fields, including gas separation,^18^ energy storage,^19^ catalysis,^20^ sensing^21,22^ and magnetic refrigeration.^23^ The unique porous structure of MOFs can accommodate randomly twisted polymer chains and enhance interfacial interactions by linker functionalization.^24^ For example, nano HKUST-1 particles were incorporated into PVA and the resultant HKUST-1/PVA nanocomposites exhibited excellent thermal stability, mechanical and UV-shielding properties.^25^ Chen *et al.*^26^ used Cu-MOF to improve the mechanical, water vapor barrier and UV-shielding properties of cellulose acetate films. Besides the thermal and mechanical properties, MOFs can also bring polymers the magnetic,^27^ and antibacterial^28^ properties. Therefore, the combination of MOFs and PI is very promising for the development of novel MOF/PI composites with enhanced mechanical properties and even elicits functions that are potentially useful in many fields. Hu *et al.*^29^ pointed out that obvious plastic deformation of PI membranes can be observed by scanning electron microscopy (SEM) and atomic force microscopy (AFM) because of the strong interactions between Cu_3_(BTC)_2_ and PI. The results observed by Zhao *et al.*^30^ showed that NH_2_-UiO-66 and ZIF-8 exhibited stronger affinity with PI membranes than MIL-53(Al). Mix-matrix membranes were shown to remove a set of six pharmaceuticals (PhACs) highly efficiently. However, few papers have been published on the neutron shielding application of MOF/PI composites. We believe this is an interesting research area, as it assists in understanding and developing a novel neutron shielding material irrespective of the packing weight used.

In this work, a rare-earth-metal gadolinium-based metal–organic framework (Gd-MOF) was synthesized from GdCl_3_ and pyromellitic dianhydride (PDMA) in *N*,*N*-dimethylformamide (DMF), namely Gd_4_(1,2,4,5-BTEC)_3_ (H_4_BTEC = 1,2,4,5-benzenetetracarboxylic acid).^31^ Subsequently, various contents of Gd-MOF were introduced into PI films by *in situ* polymerization. Vacuum pumping technology can contribute to the compact morphology of PI composites, and nanosized Gd-MOF particles can be well dispersed with a high weight filling fraction. The obtained Gd-MOF/PI composites have excellent thermal stability, mechanical strength and neutron shielding properties, showing strong potential application in shielding materials.

## 2. Experimental

### 2.1. Materials and chemicals

GdCl_3_·6H_2_O (99.9%), pyromellitic dianhydride (PMDA, 98.0%), 4,4′-oxydianiline (ODA, 98.0%), and triethylamine (99.5%) were obtained from Energy Chemical. Ethyl ether, *N*,*N*-dimethylformamide (DMF) and *N*,*N*-dimethylacetamide (DMAc) were purchased from Aladdin®. Deionized water was provided by a Ketone lab VIP machine and used in all experimental procedures. All chemicals were used as obtained without any further purification.

### 2.2. Synthesis of Gd-MOF

Using a neutron absorber, Gd-MOF containing a PMDA organic ligand identical to the PI monomer was synthesized according to previously published protocols^32^ with slight modification. First, 10 mmol GdCl_3_·6H_2_O and 7.5 mmol PMDA were stirred in 100 mL DMF. Subsequently, 5–10 g triethylamine was added dropwise with vigorous stirring until a flocculent suspension was formed. After that, the mixture was placed in a sealed Teflon-lined stainless-steel autoclave and heated for 12 h at 120 °C to form a white precipitate. After washing thoroughly with DMF, ethanol and ethyl ether, the white purified Gd-MOF precipitate was acquired and then dried at 70 °C to obtain fine particles.

### 2.3. Preparation of Gd-MOF/PI films

**Fig. 1:**
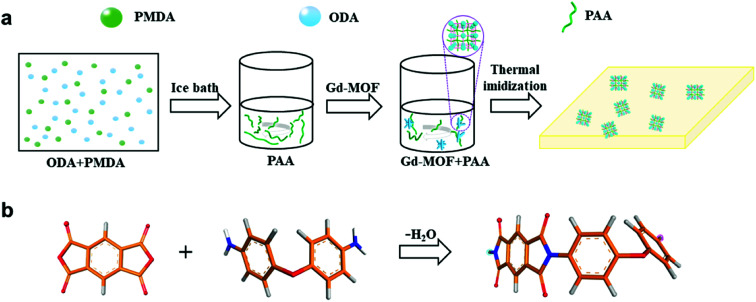
(a) Scheme of the procedure for the preparation of Gd-MOF/PI films; (b) scheme of the PI monomer. Color code for different elements: O (red), C (orange), H (gray) and N (blue).

Initially, Gd-MOFs were dispersed at different contents (0–20 wt%) in 10 mL anhydrous DMAc, which was treated for 30 min in an ultrasonic bath to form a suspension. While the Gd-MOFs were sonicating, 0.801 g ODA was added into a 50 mL three-necked flask with 20 mL anhydrous DMAc in an ice bath under a nitrogen atmosphere.^33^ PMDA (0.883 g) was subsequently added to the flask and stirred for 4 h to obtain a viscous polyamide acid (PAA) solution. During the stirring process, the prepared MOF solution was mixed with the PAA solution to appropriately increase the viscosity of the PAA solution. These Gd-MOF/PAA solutions were poured into cleaned glass dishes and heat-treated at 80 °C for 3–5 h to remove most of the solvent. Finally, the resultant films were placed in a muffle furnace at 100 °C, 150 °C, 200 °C, 250 °C and 330 °C for 1 h to obtain Gd-MOF/PI films (about 100 μm in thickness). The procedure for preparing the Gd-MOF/PI films is shown in Fig. 1.

### 2.4. Structural analysis

The structures of the Gd-MOF and Gd-MOF/PI films were analyzed by an X-ray diffractometer (XRD, X'Pert, Netherlands) operated at a voltage of 40 kV with Cu-Kα radiation. The prepared coarse fillers and Gd-MOF/PI films were ground to fine powders prior to XRD analysis.

Fourier transform infrared (FTIR) spectra were recorded on a Nicolet 6700 instrument using potassium bromide (KBr) pellets to characterize the functional groups of the Gd-MOF fillers and Gd-MOF/PI films with a resolution of 4 cm^−1^. Similarly, the products were ground to fine powders prior to FTIR analysis.

The Brunauer–Emmett–Teller (BET) surface area and porosity measurements were determined with N_2_ adsorption using a surface area analyzer (Micromeritics TriStar II 3flex, USA) at 77 K. Approximately 0.1 g Gd-MOF was outgassed in a vacuum oven at 120 °C for 8 h before the adsorption measurements, and the surface areas were estimated with the BET equation.

### 2.5. Thermal analysis

Small pieces of Gd-MOF/PI films were cut from bulk composites. These pieces and Gd-MOF particles were subjected to thermogravimetric analysis (TGA, TGA5500) at a heating rate of 10 °C min^−1^. The temperature for testing ranged from 25–800 °C with nitrogen purging gas.

Dynamic thermomechanical analysis (DMA, DMA850) was used to measure the glass transition temperature (*T*_g_) of the Gd-MOF/PI films, which were cut into rectangular shapes before testing. The *T*_g_ results correspond to the average value of 3–5 specimens.

### 2.6. Mechanical properties

The tensile properties, including tensile modulus, tensile strength and elongation at break, of the Gd-MOF/PI films were investigated according to GB/T 1040.3–2006 using computer-controlled electronic tensile testing machines (Instron 3369 and 5982) at room temperature (RT) and 400 °C, respectively. The samples were subjected to a cross head speed of 2 mm min^−1^. For each Gd-MOF/PI film, at least 3–5 identical specimens were tested, and the average results along with the standard deviation were analyzed.

Standard samples were heated in the aging test chamber (Model 401) before the tensile test to obtain thermal aging test data.

### 2.7. Morphological observations

Transmission electron microscopy (TEM) images were obtained with a microscope (FEI Tecnai G2 F20 S-Twin) to characterize the morphology of Gd-MOF. The MOF powders were dissolved in methanol and treated for 30 min in an ultrasonic bath. Then, the mixed solution was dropped on the copper mesh and subsequently observed by TEM.

Scanning electron microscopy (SEM) images were obtained with a microscope (ZEISS ΣIGMA) under a 20 kV accelerating voltage to characterize the fractured cross-sections of Gd-MOF/PI films. A knife was used to leave a suitable depth of cutting marks around the sample and then the sample was soaked in liquid nitrogen for 5–10 min until it became brittle. Finally, the samples were pulled off along the marks to obtain brittle sections. It should be noted that the Gd-MOF/PI films were coated in gold with an SBC-2 ion sputtering device for 90 s before SEM was performed.

### 2.8. Neutron shielding efficiency

SuperMC software^34^ and the ENDF/B-6.6 data library were used to evaluate the neutron shielding efficiency of fabricated Gd-MOF/PI films to investigate their application as a new neutron transport design and safety evaluation system.

**Fig. 2:**
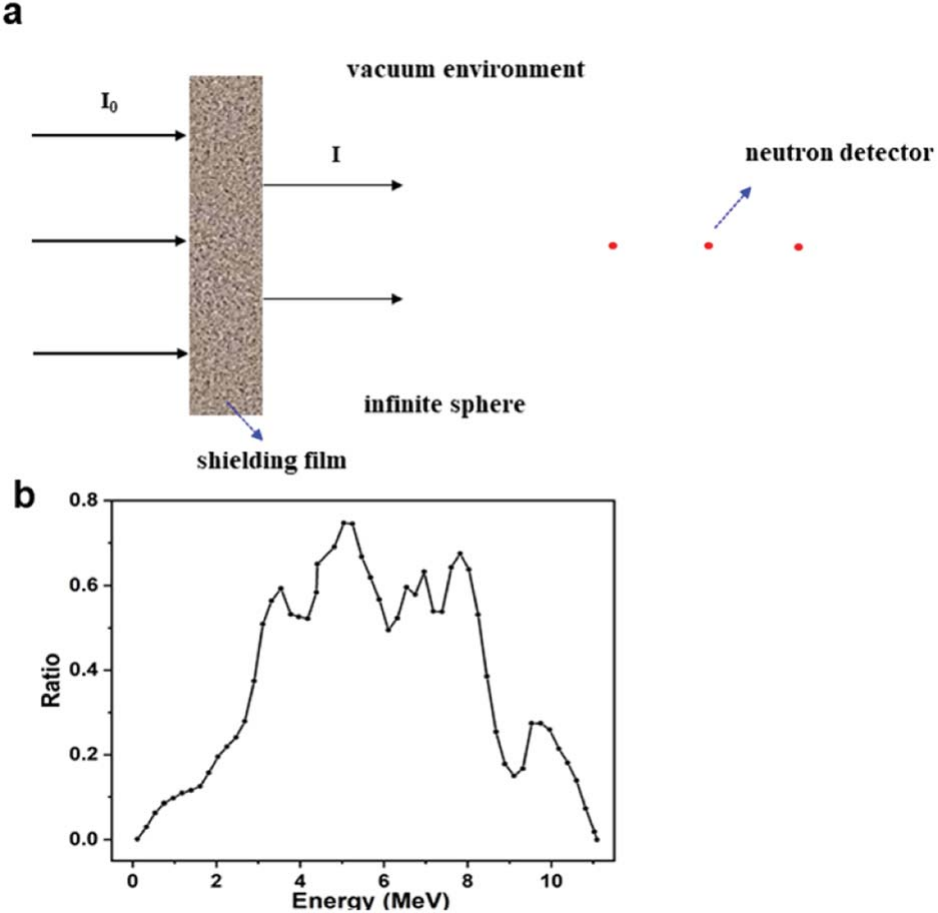
(a) Geometric model of the neutron shielding simulation experiment; (b) energy response curve of Am–Be neutron source.

As shown in Fig. 2a, the SuperMC model was used to simulate the shielding efficiency of Gd-MOF/PI films with different thicknesses (0–5 cm) and Gd-MOF contents (0–20 wt%). This model has a vacuum environment, and the four walls are total reflection surfaces, which ensures the low energy loss of neutrons during their propagation process. In this model, various energy neutrons were produced from a simulated thermal neutron source (0.0253 eV) or Am–Be source, whose energy response curve is shown in Fig. 2b. Neutron permeability *I*/*I*_0_ was calculated by eqn (1):^35^

1 *I*/*I*_0_ = *B*_*n*_ × exp(−*Σd*)

where *I*_0_ represents the neutron radiation dose without shielding films, *I* refers to the radiation dose through shielding films, *B*_*n*_ is the cumulative factor, *Σ* is the macroscopic cross-section and *d* is the thickness of shielding films.

Two cases of neutron shielding properties in Gd-MOF/PI films were investigated in this work. The first investigation examined the relationship between the Gd-MOF content and neutron shielding properties by calculating the *I*/*I*_0_ of films with different Gd-MOF contents. The elemental composition (wt%) and density (*ρ*) of Gd-MOF/PI films with different contents of Gd-MOF is shown in Table 1. The second investigation involved examining the relationship between the thickness and neutron shielding properties of Gd-MOF/PI films. The thickness of the films was varied from 0 to 0.2 mm for the thermal neutron shielding test and 0 to 5 mm for the fast neutron shielding test. Above source data (such as elemental composition, density, thickness and neutron source) were input into the SuperMC program. In order to ensure the calculation accuracy, the calculation statistical error of every case is less than 1% and the corresponding neutron shielding results would be discussed in the following paragraph. Moreover, to investigate experimental neutron shielding data, the Gd-MOF/films with same thickness (100 μm) are bonded with commercial adhesives. In this case, the relationship between the thickness and experimental neutron shielding properties can be constructed and compared with the simulated results, which will be presented in future works.

**Table 1:** Elemental composition (wt%) and density (*ρ*) of Gd-MOF/PI films with different filler contents

Gd-MOF content/wt%	C	H	O	N	Gd	Density/g cm^−3^
0	0.69110	0.02618	0.20942	0.07330	0	1.400
1	0.67666	0.02553	0.21846	0.07128	0.00807	1.387
3	0.64927	0.02431	0.23559	0.06744	0.02339	1.362
5	0.62369	0.02317	0.25159	0.06386	0.03769	1.337
7	0.59976	0.02210	0.26656	0.06051	0.05107	1.314
10	0.56661	0.02062	0.28730	0.05586	0.06961	1.280
15	0.51766	0.01843	0.31792	0.04901	0.09698	1.228
20	0.47520	0.01653	0.34448	0.04306	0.12072	1.179

## 3. Results and discussion

### 3.1. Microstructural characteristics

#### 3.1.1. Characterization of Gd-MOF fillers

**Fig. 3:**
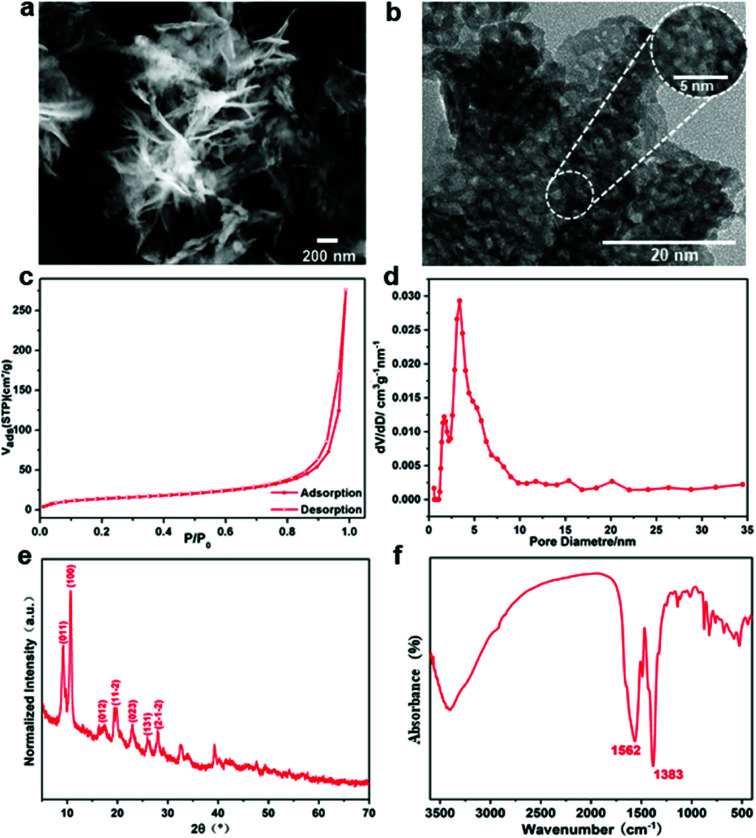
Microstructural characterization of Gd-MOF: (a and b) SEM image and TEM image; (c and d) typical isothermal nitrogen adsorption–desorption isotherm and pore-size distribution curve; (e and f) XRD pattern and FTIR spectra.

The characteristics of the fillers and their interaction with the polymer matrix are two key factors affecting their distribution in polymer composites. In this section, the morphology and crystallinity of the Gd-MOF fillers were investigated prior to the preparation of Gd-MOF/PI films. The combination of irregular flakes and needle-like shapes with a width of 100 nm and porous structures with a pore diameter of 0–5 nm were observed with SEM and TEM (Fig. 3a and b). The N_2_ adsorption–desorption isotherms of the typical IV mode (Fig. 3c) reveal that the Gd-MOF fillers have a mesoporous structure (Fig. 3d).

Gd-MOF particles are suggested to be composed of small crystallites by the broad characteristic peaks in the XRD pattern shown in Fig. 3e. All peaks are found to be in good agreement with the results described in other protocols.^32^ As shown in the FTIR spectrum of Gd-MOF (Fig. 3f), the strong band at approximately 3392 cm^−1^ shows the O–H stretching vibration of the water ligand. The peaks at 1383 cm^−1^ and 1562 cm^−1^ are assigned to the symmetric and asymmetric vibrations of carboxyl functional groups originating from PMDA, indicating that the Gd-MOF fillers were synthesized.

#### 3.1.2. Spectroscopic investigation of Gd-MOF/PI

**Fig. 4:**
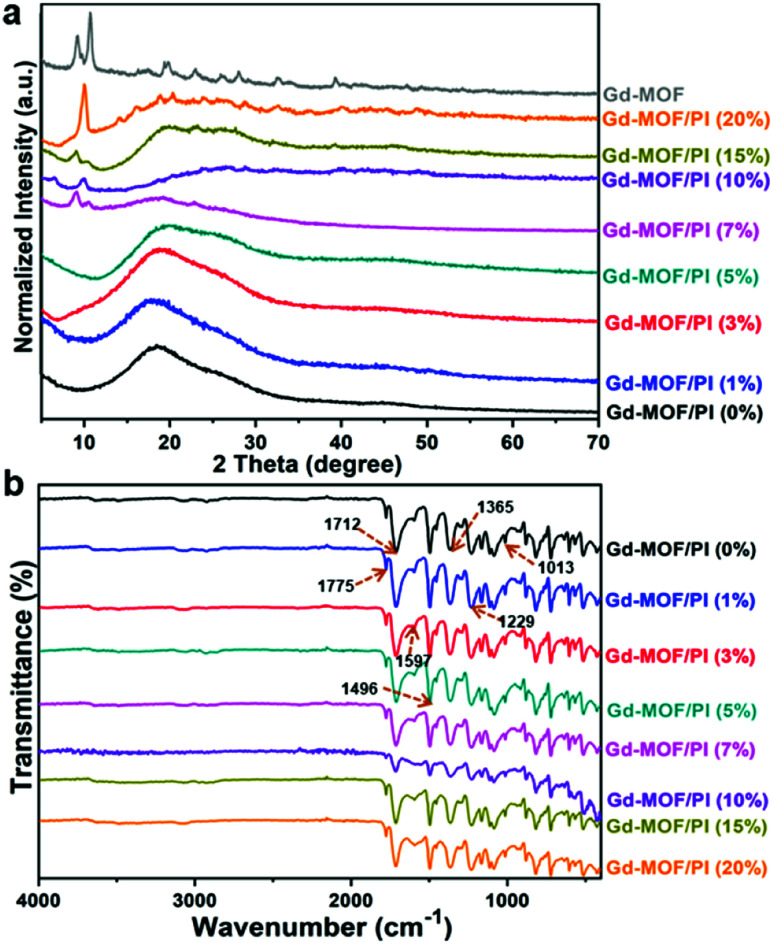
Spectroscopic characterization of Gd-MOF/PI films: (a) XRD pattern; (b) FTIR spectra.

Fig. 4a reveals the XRD patterns of Gd-MOF/PI films comprising 0–20 wt% Gd-MOF. In the case of pure PI, the pattern exhibits a broad peak reflection at approximately 19°, indicating its amorphous nature.^36^ The addition of a small number of Gd-MOF fillers (0–5 wt%) to the PI matrix also reveals roughly the same peak reflection because the filler content is too low to be detected in the XRD patterns. Nevertheless, as the filler content increases from 7 to 20 wt%, several sharp diffraction peaks appear, while the intensity of the broad peak reflection of pure PI decreases. Obviously, the existence of the characteristic signal of Gd-MOF at 2*θ* = 15–70° in the XRD pattern of Gd-MOF/PI composites (Fig. 4a) confirm the successful incorporation of Gd-MOF in to the polyimide. It is worth noting that the slight difference between the diffraction peaks of Gd-MOF and Gd-MOF/PI at 2*θ* = 5–15° may be due to the amorphous nature of polymer matrix, which can be observed in XRD results of other literature.^37^

The FTIR spectra of the Gd-MOF/PI films shown in Fig. 4b provide further evidence for the physical mixture. The peaks of C=O asymmetric (1775 cm^−1^), C=O symmetric stretch (1712 cm^−1^), and C–N stretch (1365 cm^−1^) groups of the imide ring^38^ are observed, indicating that the PI films are well prepared. The bands at 1597 cm^−1^ and 1496 cm^−1^ are the C=O stretch of amide groups and the C–N stretch of the C–N–H group, respectively. The spectra also reveal C–O asymmetric and symmetric stretches of diphenyl ether^39^ at 1229 cm^−1^ and 1013 cm^−1^, respectively. Moreover, other characteristic bands of pure PI all appear in Gd-MOF/PI films with similar relative intensities, and no obvious shift in adsorption peaks occurs. This result indicates a lack of strong chemical bonding between Gd-MOF and the PI matrix. The XRD and FTIR results show the successful fabrication of Gd-MOF/PI films. Compared with other methods,^40,41^ the fabrication process is generally simple compared because the films were formed without the packing pretreatment.

### 3.2. Thermal stability of Gd-MOF/PI

**Fig. 5:**
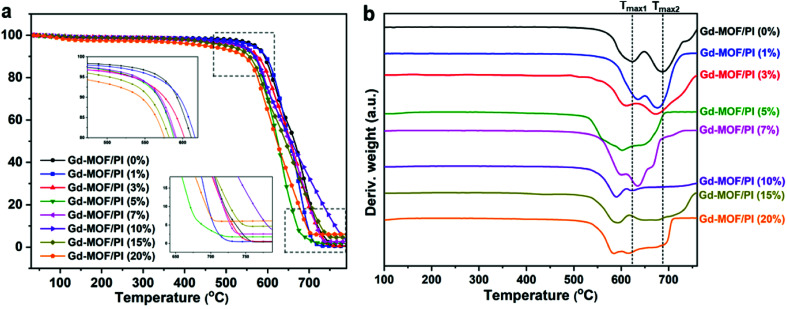
(a) TG and (b) DTG traces of Gd-MOF/PI films.

The thermal stability is crucial for the applicability of the Gd-MOF/PI films because the service temperature of the reactor components in Generation IV nuclear energy systems is relatively high. TGA was used to determine the thermal stability, with the thermogram and thermal decomposition temperature shown in Fig. 5 and Table S1,† respectively. Fig. 5a shows that all Gd-MOF/PI films exhibited similar thermal degradation trends.^42^ This result verifies that the addition of Gd-MOF has little influence on the structural integrity of the PI matrix. Additionally, the PI with higher loadings of fillers have an earlier onset of initiation temperatures for all steps in the decomposition and more residue remains with increasing Gd-MOF particle loadings (as shown in the inset of Fig. 5a).^43^ This result suggests that weak physical interactions, such as the encapsulation of Gd-MOFs by the PI matrix, somehow prevent complete decomposition. However, the decomposition temperatures of 10% (*T*_10_), 50% (*T*_50_) and maximum (*T*_max_) mass loss of Gd-MOF/PI films decrease compared with those of pure PI film. The decreased maximum decomposition temperature (*T*_max1_ and *T*_max2_) also can be showed in the Fig. 5b. These results are due to the lower complete decomposition temperature of Gd-MOF, as shown in Fig. S2.†

**Fig. 6:**
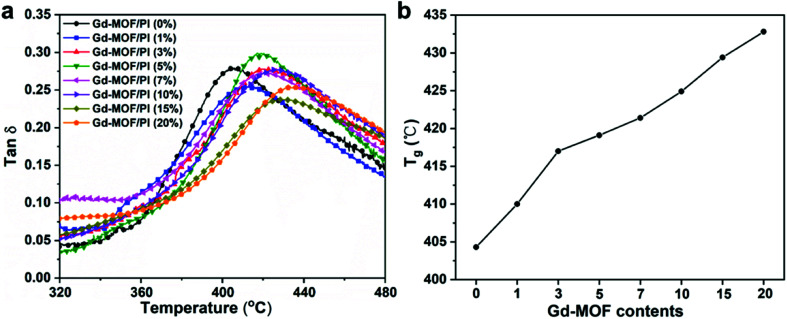
Thermal stability of Gd-MOF/PI films: (a) DMA curves; (b) *T*_g_*vs.* Gd-MOF contents.

The DMA data of Gd-MOF/PI films are shown in Fig. 6a, where the abscissa values at the apex of curves represent the *T*_g_. Compared with the *T*_g_ of pure PI (404.3 °C) that much higher than the reported result (225.0 °C),^44^ the *T*_g_ of Gd-MOF/PI containing 20 wt% Gd-MOF increases by 7.2% (Fig. 6b). Generally, the *T*_g_ of polymer composites may be affected by several factors, such as the particle–matrix interface area, polymer tactility, molecular weight and cross-linking density.^45^ In this study, the improvement of *T*_g_ may be attributed to the decline in free volume in films,^46^ consequently restricting the thermal movement of chain segments *via* the encapsulation of Gd-MOF.^47^ As a result, polymer chains need to absorb more energy to overcome the energy barrier, which macroscopically results in an increase in *T*_g_.

### 3.3. Mechanical properties and reinforcement mechanism of Gd-MOF/PI

**Fig. 7:**
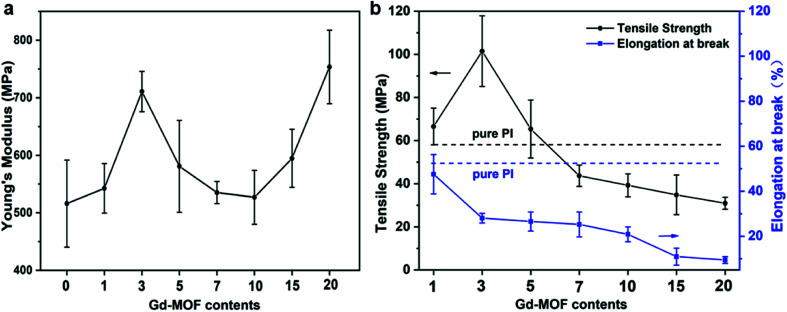
Mechanical properties of Gd-MOF/PI films: (a) Young's modulus; (b) tensile strength and elongation at break.

Based on the above results, the mechanical properties of Gd-MOF/PI films were then investigated. As shown in Fig. 7a, the Young's modulus of Gd-MOF/PI films increased first with the content of Gd-MOF ranging from 0 wt% to 3 wt%, which is attributed to good dispersion and interfacial bonding between the Gd-MOF particles and PI matrix, as evidenced by DMA. When the Gd-MOF content is greater than 3 wt%, the gradually decreased bonding strength originating from the agglomeration of Gd-MOF particles results in a decrease in the Young's modulus. Furthermore, the Young's modulus increases again with the content of Gd-MOF ranging from 10 to 20 wt%, and the maximum increase (−46.0%) is observed for the 20 wt% filler content compared with that of the pure PI film. As shown in Fig. S3,† the microstructures of Gd-MOF/PI films with 10 and 15 wt% Gd-MOF have completely changed compared with that of the other Gd-MOF/PI films. The agglomeration of Gd-MOF particles with higher Young's modulus become the main component of the PI composites. The interaction between these aggregates strengthens the original bond between the polymer chains and reduce the free volume of the PI composites.^48^

**Fig. 8:**
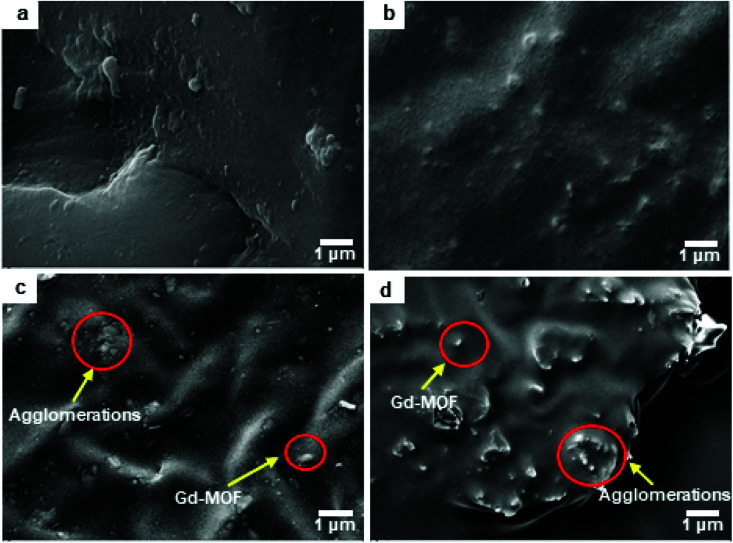
SEM micrograph surface of Gd-MOF/PI films: (a) 1 wt%, (b) 3 wt%, (c) 5 wt% and (d) 10 wt% Gd-MOF contents.

The effects of fillers on the tensile strength and elongation at break of Gd-MOF/PI films are presented in Fig. 7b. The tensile strength of pure PI (84.8 MPa) is approximately consistent with the previous results (44.1–90.0 MPa),^49^ and the elongation at break (35.8%) is higher than the corresponding results (6.0–30.0%).^49^ The addition of Gd-MOF obviously enhances the tensile strength, especially when the filler content is lower than 3 wt%, while it decreases the elongation at break of the Gd-MOF/PI films. The maximum increase in the tensile strength (−74.6%) is observed for the Gd-MOF/PI film containing 3 wt% Gd-MOF compared with that of pure PI. Further, as shown in Fig. 8a and b, the SEM images of the Gd-MOF/PI films with either 1 or 3 wt% fillers display a very smooth and flat surface without any obvious matrix imperfections. Additionally, Gd-MOF fillers exhibit good dispersion and low surface roughness due to the good interfacial interaction between fillers and the PI matrix. The porous structure of the Gd-MOFs can provide a platform for the interlacement of PI chains and the organic PMDA surfaces and create a good compatible interface with the PI matrix, resulting in efficient interfacial load transfer. Moreover, Gd-MOF/PI films with 5 or 10 wt% Gd-MOF can be observed in Fig. 8c, d and S3,† and the filler–matrix interaction of these films causes a decrease in tensile strength. The incorporation of high filler content into the PI matrix results in a high surface density of micro-scale agglomerated Gd-MOF fillers, which increases the matrix defects, and consequently, the surface roughness and stress concentration are expected to increase.^50^

**Fig. 9:**
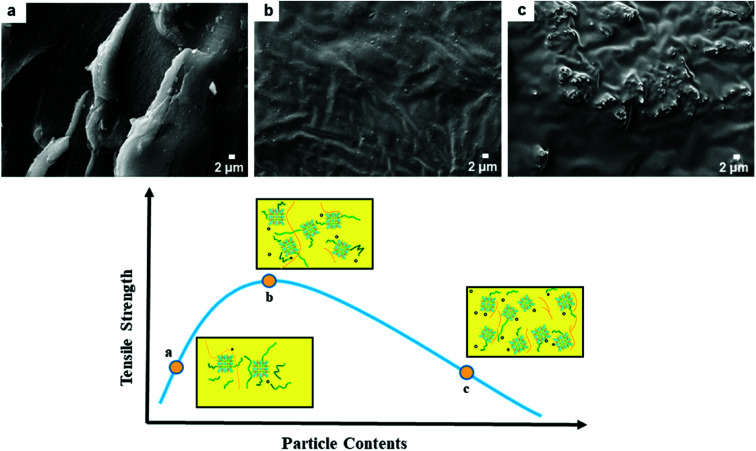
Reinforcement mechanism of Gd-MOF in PI: the holes and cracks are represented by open circles and orange curves, respectively; PI polymer chains are represented by green lines surrounding the Gd-MOFs.

Similar phenomena are also observed in other PI composites,^51,52^ which prompts us to explore the underlying reinforcement mechanism. Microscopically, the tensile strength of the Gd-MOF/PI films is affected by two factors: interfacial stress conduction and the presence of defects (Fig. 9). As shown in Fig. 9a, the Gd-MOFs are well dispersed in the PI matrix when the filler content is relatively low. The good interfacial interaction in Gd-MOF/PI guarantees efficient interfacial stress transfer^53^ and there are few holes and cracks in the PI matrix, which mainly contribute to the improvement of tensile strength. With increasing Gd-MOF content, more defects are introduced into the Gd-MOF/PI films, which may generate more stress concentrations and microcracks under external stress.^54^ As the Gd-MOF content increases to 3%, these two effects achieve a state of equilibrium, making the tensile strength reach its peak (Fig. 9b). With a further increase in the Gd-MOF content, the number of cracks aggregates into obvious defects (Fig. 9c),^55^ leading to a stress concentration in the vicinity of the particle–polymer interface and a decrease in the tensile strength.

The elongation at break of the Gd-MOF/PI films decreases gradually with increasing Gd-MOF content. This phenomenon is due to the interfacial interaction between the Gd-MOFs and PI, which increases the number of cross-links and restricts the sliding motion of the polymer chains during the stretching process. In addition, the mechanical properties and aging results at 400 °C are shown in Fig. S4 and Tables S2, S3.† The Gd-MOF/PI film with a 1 wt% content of Gd-MOF has better thermal aging properties and mechanical properties at 400 °C. The Gd-MOF/PI film with a 3 wt% content of Gd-MOF has a better Young's modulus and mechanical properties at RT. The Gd-MOF/PI film with 1 wt% filler content is more suitable for utilization at high temperatures.

### 3.4. Neutron shielding properties

To evaluate the potential application of Gd-MOF/PI films, it is necessary to investigate the neutron shielding properties of films with different thicknesses. The thermal and fast neutron shielding properties of 1 and 3 wt% Gd-MOF/PI films were simulated by SuperMC software and shown in Tables 2 and 3, respectively. The ability to shield thermal neutrons with the same thickness improves when the Gd-MOF content increases from 1 wt% to 3 wt%. This improvement is due to the increase in the number of ^155^Gd and ^157^Gd nuclei, which results in more interactions with neutrons and higher thermal neutron attenuation of the Gd-MOF/PI films. In contrast, the fast neutron shielding ability of films with the same thickness slightly decreases as the Gd-MOF content increases. This trend occurs because hydrogen atoms are able to slow down fast neutrons, and the hydrogen content decreases with the addition of Gd-MOFs, leading to fewer interactions of neutrons with hydrogen atoms.

**Table 2:** Thermal neutron permeability *I*/*I*_0_ for 1 and 3 wt% Gd-MOF/PI films with different thicknesses

Film thickness (cm)	Thermal neutron permeability *I*/*I*_0_ (%)	
	1 wt% Gd-MOF/PI films	3 wt% Gd-MOF/PI films
0	100	100
0.001	29.6	24.9
0.01	17.2	12.5
0.025	12.5	8.0
0.05	9.4	5.0
0.075	7.5	3.5
0.1	6.3	2.5
0.125	5.4	1.9
0.15	4.7	1.4
0.175	4.1	1.1
0.2	3.6	0.9

**Table 3:** Fast neutron permeability *I*/*I*_0_ of 1 and 3 wt% Gd-MOF/PI films with different thicknesses

Film thickness (cm)	Fast neutron permeability *I*/*I*_0_ (%)	
	1 wt% Gd-MOF/PI films	3 wt% Gd-MOF/PI films
0	100	100
0.2	20.0	20.1
0.4	17.0	17.2
0.6	15.6	15.7
0.8	14.7	14.7
1	13.8	14.0
2	11.8	12.0
3	10.7	10.9
4	10.0	10.1
5	9.4	9.6

Another neutron shielding property is the relationship between the thickness of films and neutron shielding properties. With increasing thickness, both the thermal and fast neutron permeabilities both decrease significantly, indicating the improvement in the neutron shielding properties. Similar patterns can be observed in the thermal and fast neutron shielding properties of Gd-MOF/PI films with other filler contents (Fig. S5†). Moreover, the neutron shielding properties of the Gd-MOF/PI films were better than those of traditional B_4_C/PI films and borated polyethylene (Fig. S6†). Moreover, Table 4 summarizes the polyimide-based neutron shielding materials, focusing on the decomposition temperatures of different mass loss (take 5% (*T*_5_) for example), tensile strength, neutron permeability of PI-based films. It is observed that Gd-MOF/PI films possess good thermal stability and neutron shielding properties compared with the other reported PI-based composites.

**Table 4:** The decomposition temperature of different mass loss, tensile strength, neutron permeability of polyimide-based neutron shielding materials

Shielding materials	Decomposition temperature	Tensile strength	Neutron permeability	Author, year, reference
^10^B_2_O_3_ (0.5 wt%)/polyimides	282 °C (5% mass loss)	426 MPa		Mülazim *et al.*, 2011,^56^
Carborane polyimides	520 °C (5% mass loss)			Xing *et al.*, 2014,^57^
Carbon-fiber reinforced Sm_2_O_3_ (21 wt%)/polyimides	300 °C (1% mass loss)	200 MPa	50% (1 cm thickness)	Wang *et al.*, 2015,^58^
h-BN (3 wt%)/Gd_2_O_3_ (3 wt%)/polyimides		78 MPa	70% (1 cm thickness)	Baykara *et al.*, 2020,^59^
Gd-MOF (3 wt%)/polyimides	568 °C (10% mass loss)	75 MPa	14% (1 cm thickness)	Our results

As a whole, the Gd-MOF/PI films with 1 and 3 wt% contents of Gd-MOF exhibit good neutron shielding properties and have great potential to be used as effective neutron shielding materials.

## 4. Conclusions

In summary, neutron shielding materials based on Gd-MOF/PI films with various Gd-MOF contents were developed. Gd-MOF fillers play an important role in the thermal, mechanical and neutron shielding properties of Gd-MOF/PI films. The results of thermogravimetric analysis show that the glass transition temperature of Gd-MOF/PI films can be significantly enhanced by the addition of Gd-MOF due to the decline in free volume in films. In addition, the interfacial interaction improves the Young's modulus and tensile strength of the Gd-MOF/PI films, among which Gd-MOF works effectively as an active absorber to shield neutrons. Moreover, the results of the SuperMC simulation show that increasing the Gd-MOF content from 0 to 20 wt% can improve the thermal neutron shielding ability but slightly reduce the fast neutron shielding ability. The Gd-MOF/PI films with 3 wt% Gd-MOF content exhibit a high glass transition temperature of 417 °C, a relatively high elongation at break of 28.1%, and a thermal neutron permeability of 0.9% (0.2 cm in thickness). It is anticipated that the type of Gd-MOF/PI film has the potential to be used as a neutron shielding material owing to its good thermal stability, mechanical properties and neutron properties.

## Conflicts of interest

The authors declare no competing financial interests.

## Supplementary Material

RA-011-D1RA07500D-s001
