# Correction: Thermal, mechanical investigation and neutron shielding analysis for Gd-MOF/polyimide materials

**DOI:** 10.1039/d2ra90010f

**Published:** 2022-02-16

**Authors:** Chen Hu, Qunying Huang, Yutao Zhai

**Affiliations:** Institute of Nuclear Energy Safety Technology, Hefei Institutes of Physical Science, Chinese Academy of Sciences Hefei Anhui 230031 China; University of Science and Technology of China Hefei Anhui 230027 China hu2017@mail.ustc.edu.cn

## Abstract

Correction for ‘Thermal, mechanical investigation and neutron shielding analysis for Gd-MOF/polyimide materials’ by Chen Hu *et al., RSC Adv.*, 2021, **11**, 40148–40158. DOI: 10.1039/D1RA07500D.

The authors regret that the grant number quoted in the original acknowledgements section was incorrect. The correct acknowledgments section is given here:

This work was supported by the National Key Research and Development Program of China [Grant numbers 2020YFB1901900]; and the “115” Industrial Innovation Team of Anhui Province.

The Royal Society of Chemistry apologises for these errors and any consequent inconvenience to authors and readers.

## Supplementary Material

