# Supplementary material for: Thermal, mechanical investigation and neutron shielding analysis for Gd-MOF/polyimide materials
Source: RSC Adv. 2021 Dec 22;11(63):40148–58. doi: 10.1039/d1ra07500d (PMC8693875; doi:10.1039/d1ra07500d)
Supplement: RA-011-D1RA07500D-s001 [file RA-011-D1RA07500D-s001.pdf]

Electronic Supplementary Material (ESI) for RSC Advances.
This journal is © The Royal Society of Chemistry 2021

Supplementary Information

Thermal, mechanical investigation and neutron shielding analysis for Gd-MOF/polyimide materials

Chen Hu [a,b] *, Qunying Huang [a], Yutao Zhai [a]

[a]*Institute of Nuclear Energy Safety Technology, Hefei Institutes of Physical Science, Chinese Academy of Sciences, Hefei, Anhui, 230031, China*

[b]*University of Science and Technology of China, Hefei, Anhui, 230027, China*

* *Corresponding author:* hu2017@mail.ustc.edu.cn

Section 1. Supporting results

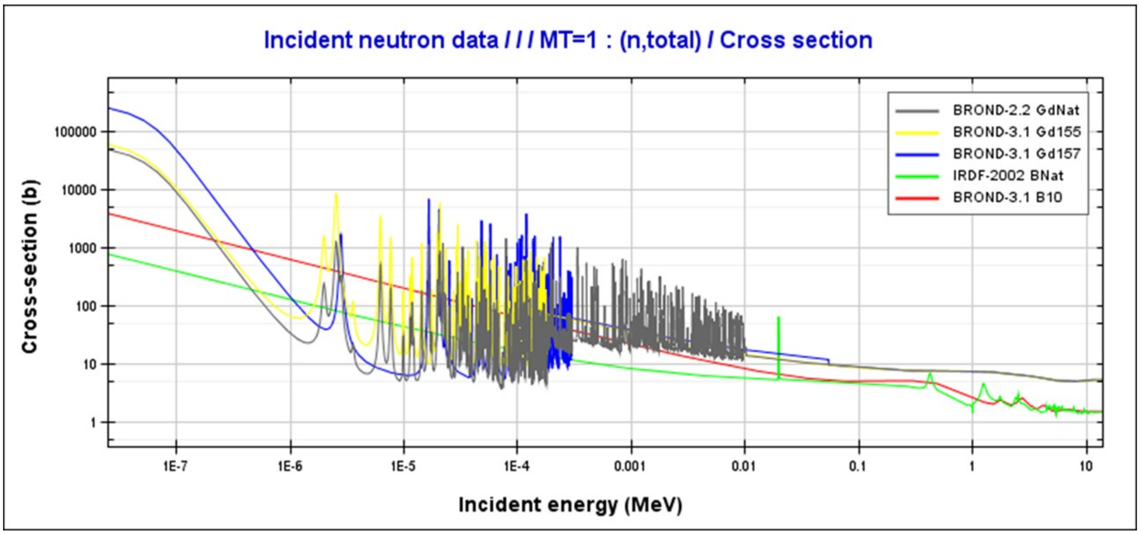

Fig. S1 Neutron absorption cross section of gadolinium and boron

Table S1 TG data of Gd-MOF/PI films

Filler content (wt %)	T_{10} (°C)	T_{50} (°C)	T_{max} (°C)	residue (%)
0	592.5	664.5	769.9	0.407
1	575.8	655.1	767.0	0.636
3	568.0	624.3	748.2	1.802
5	570.5	651.7	747.3	2.578
7	568.6	656.4	767.5	3.858
10	555.8	642.7	761.2	4.676
15	544.3	622.4	723.2	8.669
20	526.1	608.9	709.2	11.191

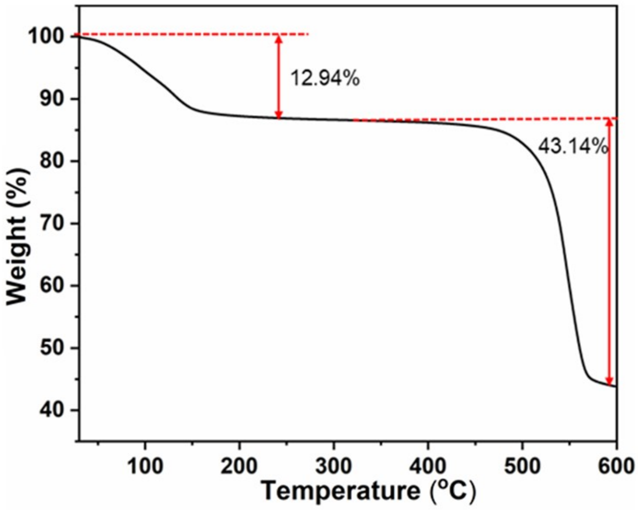

Fig. S2 TG traces Gd-MOF

As shown in Fig. S2, the TGA curve of Gd-MOF showed three stages of mass loss. The mass loss below 150 °C is due to the evaporation of water on the grafted surface and the physically absorbed water in the pores of Gd-MOF. In the temperature range of 450~550 °C, Gd-MOF is divided into metal parts and organic parts [1]. The organic part cannot withstand at this high temperature and undergo decomposition. This results in a clear downward trend of the curve. However, when temperature reaches 550 °C, the inorganic part remains as such and the decomposition process somewhat keeps stabilization.

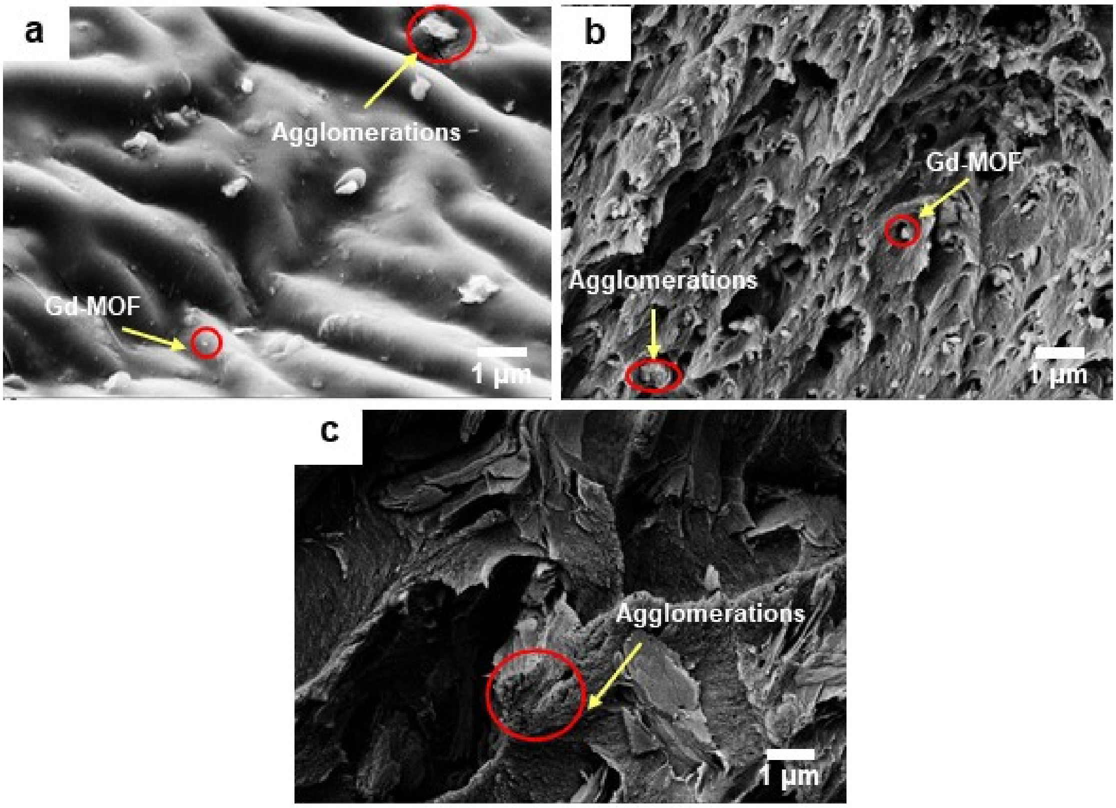

Fig. S3 SEM micrograph surface of Gd-MOF/PI films: (a) 7 wt%, (b) 15 wt% and (c) 20 wt% Gd-MOF contents.

Section 2. Mechanical properties and ageing effect at 400 ℃ on Gd-MOF/PI films

Considering that the Gd-MOF/PI films are used at high temperature, their tensile properties and the aging results of Gd-MOF/PI films at 400 ℃ were investigated. As shown in Fig. S4, the tensile strength and elongation at break decrease gradually with the increase of Gd-MOF content. Compared with that of pure PI, the maximum increase in the tensile strength (~181.3%) at 400 ℃ is observed for the Gd-MOF with a 1 wt% content. Physical aging involves the simultaneous reduction of free volume and conformational changes of molecular structure during thermo-oxidative degradation [2]. As shown in Table. S2 and Table. S3, the values of the tensile strength and elongation at break both decrease with the increase of aging time, which may be due to the limited chain scission [3]. However, the relevant mechanical properties of Gd-MOF/PI film with 1 wt% filler content are always better than that of pure PI [4], indicating the reinforcement effect of Gd-MOF in Gd-MOF/PI films. The mechanical properties of Gd-MOF/PI films with Gd-MOF content ranging from 3 wt% to 20 wt% hardly be detected owing to their brittleness at high temperature.

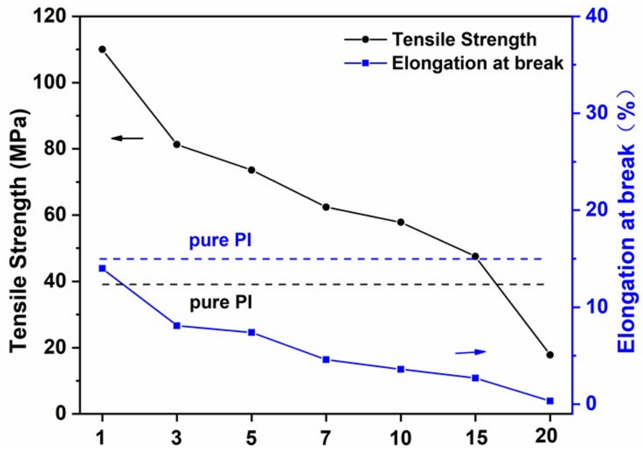

Fig. S4 Tensile strength and elongation at break of Gd-MOF/PI films at 400 ℃

Table S2 Tensile strength and elongation at break of Gd-MOF/PI films aged at 400 ℃ for 15 days

Filler content (wt%)	Tensile strength (MPa)	Elongation at break (%)
0	29.0	14.0
1	40.1	17.0

Table S3 Tensile strength and elongation at break of Gd-MOF/PI films aged at 400 °C for 30 days

Filler content (wt%)	Tensile strength (MPa)	Elongation at break (%)
0	26.0	9.8
1	28.8	12.6

Section 3. Neutron shielding properties of Gd-MOF/PI films and other polyimide composites

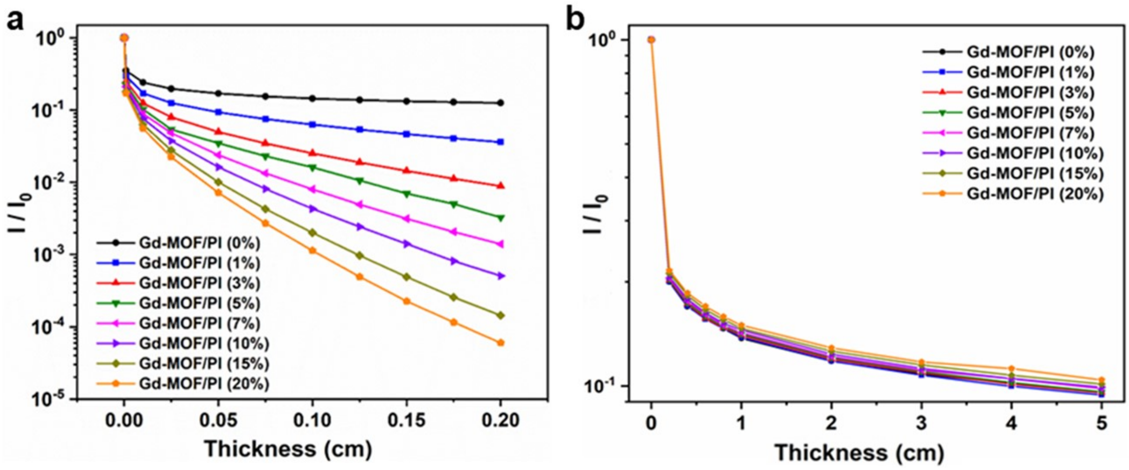

Fig. S5 Neutron permeability of Gd-MOF/PI films under different neutron sources: (a) thermal neutron source; (b) Am-Be neutron source

The thermal neutron permeability of Gd-MOF/PI and B_4C/PI film with 20 wt% content of fillers under thermal neutron source is showed in Fig. S5a. When the thickness ranges from 0 to 0.2 cm, the Gd-MOF/PI films have better thermal neutron shielding properties than those of B_4C/PI owing to the higher neutron capture cross section of Gd than B. The fast neutron permeability of Gd-MOF/PI and borated polyethylene with 3 wt% content of fillers under Am-Be source is showed in Fig. S5b. When the thickness ranges from 0 to 4 cm, the Gd-MOF/PI has better fast neutron shielding properties than borated polyethylene.

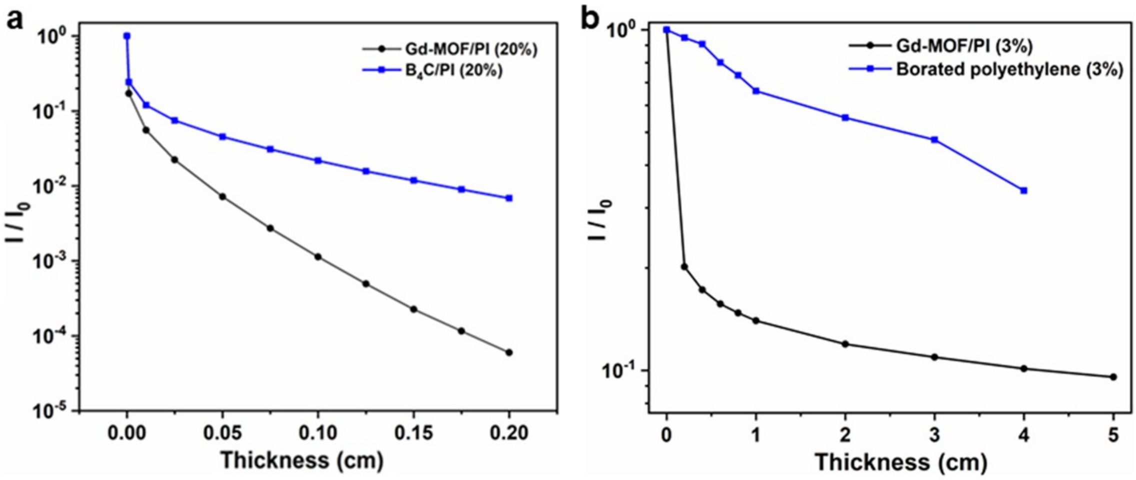

Fig. S6 Neutron permeabilities of Gd-MOF/PI films and other polymer-based composites: (a) B_4C/PI films under thermal neutron source; (b) borated polyethylene under Am-Be source

Notes and references:

[1] Wang N, Zhang Y, Chen J et al (2017) Dopamine modified metal-organic frameworks on anti-corrosion properties of waterborne epoxy coatings, Prog Org Coat 109:126-134.

[2] Odegard GM, Bandyopadhyay A (2011) Physical aging of epoxy polymers and their composites, J Polym Sci Part B: Polym Phys 49:1695-1716.

[3] Yang Y, Xian G, Li H (2015) Thermal aging of an anhydride-cured epoxy resin[J]. Polymer Degradation and Stability, Polym Degrad Stab 118:111-119.

[4] The MM, Liao K (2002) Effects of environmental aging on the mechanical properties of bamboo-glass fiber reinforced polymer matrix hybrid composites, Composites Part A 33:43-52.
